# Escherichia coli Genomic Diversity within Extraintestinal Acute Infections Argues for Adaptive Evolution at Play

**DOI:** 10.1128/mSphere.01176-20

**Published:** 2021-01-06

**Authors:** Antoine Bridier-Nahmias, Adrien Launay, Alexandre Bleibtreu, Mélanie Magnan, Violaine Walewski, Jérémie Chatel, Sara Dion, Véronique Robbe-Saule, Olivier Clermont, Françoise Norel, Erick Denamur, Olivier Tenaillon

**Affiliations:** a Université de Paris, IAME, UMR 1137, INSERM, Paris, France; b AP-HP, Sorbonne Université, Hôpitaux Universitaires Pitié-Salpêtrière-Charles Foix, Service de Maladies Infectieuses et Tropicales, Paris, France; c Institut Pasteur, Unité de Biochimie des Interactions Macromoléculaires, Paris, France; d CNRS UMR3528, Paris, France; e AP-HP, Laboratoire de Génétique Moléculaire, Hôpital Bichat, Paris, France; University of Wisconsin—Madison

**Keywords:** *Escherichia coli*, bacteria, evolution, genomes, infection

## Abstract

Adaptive processes in chronic bacterial infections are well described, but much less is known about the processes at play during acute infections. Here, by sequencing seven randomly selected isolates per patient, we analyzed Escherichia coli populations from three acute extraintestinal infections in adults (meningitis, pyelonephritis, and peritonitis), in which a high-mutation-rate isolate or mutator isolate was found. The isolates of single patients displayed between a few dozen and more than 200 independent mutations, with up to half being specific to the mutator isolate. Multiple signs of positive selection were evidenced: a high ratio of nonsynonymous to synonymous mutations (*K_a_*/*K_s_* ratio) and strong mutational convergence within and between patients, some of them at loci well known for their adaptive potential, such as *rpoS*, *rbsR*, *fimH*, and *fliC*. For all patients, the mutator isolate was likely due to a large deletion of a methyl-directed mismatch repair gene, and in two instances, the deletion extended to genes involved in some genetic convergence, suggesting potential coselection. Intrinsic extraintestinal virulence assessed in a mouse model of sepsis showed variable patterns of virulence ranging from non-mouse killer to mouse killer for the isolates from single patients. However, genomic signature and gene inactivation experiments did not establish a link between a single gene and the capacity to kill mice, highlighting the complex and multifactorial nature of the virulence. Altogether, these data indicate that E. coli isolates are adapting under strong selective pressure when colonizing an extraintestinal site.

**IMPORTANCE** Little is known about the dynamics of adaptation in acute bacterial infections. By sequencing multiple isolates from monoclonal extraintestinal Escherichia coli infections in several patients, we were able to uncover traces of selection taking place at short time scales compared to chronic infection. High genomic diversity was observed in the patient isolates, with an excess of nonsynonymous mutations, and the comparison within and between different infections showed patterns of convergence at the gene level, both constituting strong signs of adaptation. The genes targeted were coding mostly for proteins involved in global regulation, metabolism, and adhesion/motility. Moreover, virulence assessed in a mouse model of sepsis was variable among the isolates of single patients, but this difference was left unexplained at the molecular level. This work gives us clues about the E. coli lifestyle transition between commensalism and pathogenicity.

## Abstract (precis)

Little is known about the dynamics of adaptation in acute bacterial infections. By sequencing multiple isolates from monoclonal extraintestinal Escherichia coli infections in several patients, we were able to uncover traces of selection taking place at short time scales compared to chronic infection.

## INTRODUCTION

With environmental changes, microorganisms are facing new challenges and new opportunities for adaptation. Besides laboratory experiments (1, 2), assessing bacterial adaptation in the wild during environmental changes is challenging, particularly in a medical context. Most of the available data to date come from chronic infections such as lung colonization and infection of cystic fibrosis patients by environmental bacteria such as Burkholderia dolosa (3) and Pseudomonas aeruginosa (4–6). In these processes, which may last for years, multiple studies have revealed proof of molecular adaptation, and three features of these adaptations are particularly striking. (i) As these patients are heavily treated with antibiotics, multiple antibiotic resistances have been found to emerge in these bacterial populations over the years. (ii) Among these bacterial populations, a large fraction of high-mutation-rate clones named mutators can be found. These mutators are bacteria that have undergone inactivation of some DNA repair systems, and they subsequently have elevated mutation rates (7). The most common defect found among natural mutator isolates is an impairment of methyl-directed mismatch repair (MMR), a postreplication error correction system relying on the action of genes *mutS*, *mutL*, and *mutH* (8). Experimental evolution (9), simulations (10), and theoretical analyses have shown that an increase in the mutation rate can be selected for when the supply of beneficial mutations is large (second-order selection). In these cases, the excess of beneficial mutations produced by the mutator clones drives them to a high frequency within the population, and the presence of a mutator allele can therefore be considered a signature of sustained adaptation. (iii) Genomic studies of epidemic bacterial clones and experimentally evolved laboratory strains (11) have revealed convergence at a molecular level (12, 13) that can be seen as a hallmark of adaptation. Among all the possible mutations that may occur, finding mutations with comparable effects reflects the filtering action of natural selection. For instance, in *Burkholderia* infections (3), aside from convergence in antibiotic resistance genes, recurrent mutations in the respiration pathway were found, implying specific adaptation to the respiratory tract environment. Similarly, in P. aeruginosa, convergence was observed in genes involved in cell wall/lipopolysaccharide (LPS)/capsule production, secreted factors, and transcription factors (5).

All these observations prove that adaptation is at play in chronic infections. Indeed, these infections offer the best opportunity for adaptation to proceed: population sizes are large, and the time scale is long, with both factors giving opportunities for a mutation to emerge and be selected for. One question that remains open is whether acute infections that have a much shorter time scale are also coupled to adaptation.

Extraintestinal pathogenic Escherichia coli (ExPEC) can be used as a model opportunistic pathogen responsible for acute infections compared to the chronic infections cited above. E. coli is a versatile species, being a commensal of the gut of vertebrates, the dominant facultative anaerobe of the human microbiota (14), and a life-threatening pathogen responsible for about 1 million deaths per year due to intestinal and extraintestinal pathologies (15). Extraintestinal infections caused by E. coli are mainly urinary tract infection (UTI), suppuration, septicemia, and, less often, newborn meningitis. The intrinsic extraintestinal virulence of the strains can be assessed in a mouse model of sepsis (16) that shows its multigenic character (17), with an important part being played by the iron capture system (18, 19).

The literature contains some hints that adaptation may occur during acute extraintestinal infections by E. coli. (i) In an early study, isolates from a monoclonal UTI displayed a variety of colony morphologies (20). More recently, when sampling multiple isolates from 19 E. coli extraintestinal infections, Levert and colleagues recovered traces of genetic heterogeneity associated with phenotypic diversity within isolates of a single clone. Indeed, isolates from the same clonal infection differed in their antibiotic resistance, growth rate, and stress resistance; these changes resulted in differences in their intrinsic virulence in a mouse model of sepsis (21). (ii) Moreover, in the study by Levert et al. and, more broadly, in UTI, mutator strains are recovered more frequently than commensal strains (21, 22) and can even sometimes dominate the population (23). Two mechanisms of adaptation to UTI conditions were observed in animal models: Labat et al. showed that a mutator strain could quickly evolve to avoid clearance and establish chronic infections (24), and Cooper et al. showed that deletion of MMR genes was selected for during a 48-h ascending UTI, presumably due to a higher switching rate of motility expression in MMR-deficient strains (25). (iii) Finally, within-household adaptive evolution of the E. coli sequence type 131 (ST131)-H30 pandemic clonal group has been observed in pathological samples from two family members presenting severe extraintestinal infections (26).

In addition to this evidence based on diversity within an infection or during short-term evolution in animal models, epidemiological studies suggest the recruitment of pathoadaptive mutations during acute infections (27). The pioneering work of Sokurenko and colleagues (28) used comparative genomics to identify the signature of selection in the type 1 fimbria component-encoding gene *fimH*. They found this gene to be enriched in nonsynonymous variants in UTI isolates. Experimental studies (29) confirmed the role of these mutations in modulating adhesion affinity/specificity and revealed their contribution to pathology in a mouse model of UTI.

All the keys for adaptation seem to be present in E. coli acute extraintestinal infections: genetic diversity associated with some phenotypic diversity having consequences on virulence, proof that adaptation is possible in animal models, and candidate genes that seem to contribute to the adaptive process. To obtain more insights into the processes at play in these infections, we characterized thoroughly the genomic diversity present within the infective populations of 3 adult patients sampled from the 19 infections mentioned above (21). We chose to study these 3 patients (out of 11 monoclonal infections tested for mutation rates [21; E. Denamur, unpublished data]) in whom strong mutators were found, as their presence is indirect evidence for ongoing selection. One patient (patient 11) was sampled from a rare case of adult meningitis following neurosurgical procedures, one patient (patient 13) suffered from pyelonephritis, and one patient (patient 17) presented with peritonitis.

## RESULTS AND DISCUSSION

**FIG 1:**
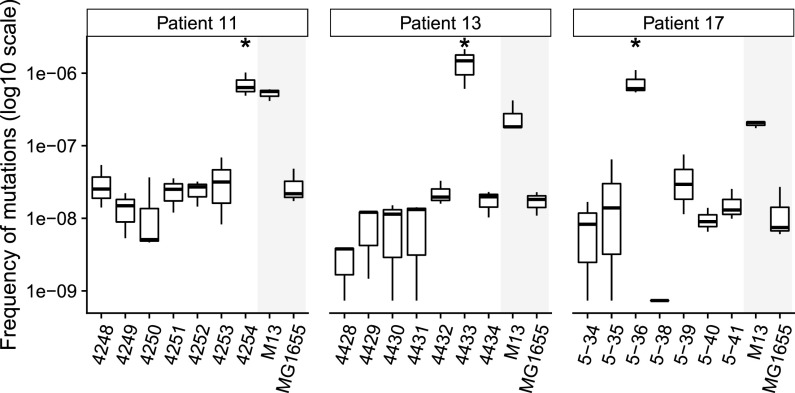
Mutation rates of the E. coli isolates from the three patients. The *y* axis represents the frequency of rifampicin resistance acquisition expressed as the median of at least nine values corresponding to three independent cultures. E. coli M13 and K-12 MG1655 are mutator (*mutS*-deleted) and nonmutator controls, respectively, and are shaded in gray. * indicates a mutator.

Among the previously characterized collection of E. coli extraintestinal infections (21), we selected three patients who were infected by a unique clone presenting genetic microheterogeneity (based on pulsed-field gel electrophoresis and/or enterobacterial repetitive intergenic consensus PCR) as well as a strong mutator (>50-fold increase in mutagenesis). The epidemiological characteristics of the isolates are presented in Table 1. We sequenced seven isolates randomly selected from each patient. Mutation rate estimates of the isolates sampled in each of these three patients are reported in Fig. 1. In each infection, a single strong mutator (14% of the isolates) is easily distinguishable; indeed, isolates 5-36 of patient 17, 4433 of patient 13, and 4254 of patient 11 present a highly elevated mutation rate, comparable to the rate estimated from our control MMR-deficient strain M13 (Fig. 1).

**TABLE 1:** Main characteristics of the E. coli strains studied^*a*^

Patient	Type of infection	Phylogroup	ST^WU^	ST^IP^	Serotype	*fimH* type
11	Meningitis	A	1434	817	O18:H14	53
13	Pyelonephritis	B2	372	40	O83:H31	211
17	Peritonitis	A	4358	132	O25:H16	54

a ST^WU^ and ST^IP^ are sequence types determined using schemes from Warwick University Medical School and Institut Pasteur, respectively.

### Isolate description and within-sample diversity.

Looking at the whole genome, the isolates from the three infections display different levels of diversity, with a combination of point mutations (single nucleotide polymorphisms [SNPs] and small insertions and deletions [indels]) and large deletions (Table 2). The isolates from the cerebrospinal fluid of patient 11 displayed the lowest level of diversity with 36 mutations overall, with 17 (47%) being specific to the mutator. The isolates from pyelonephritis of patient 13 displayed a total of 65 independent mutations, of which 22 (34%) are specific to the mutator. Finally, the isolates from patient 17, who suffered from peritonitis, displayed a total of 214 independent mutations, with the mutator accounting for 48% of them (103 mutations). The extensive diversity suggests two hypotheses. The first one is that the divergence among isolates started a long time ago and predated the infection. Indeed, when considering a spontaneous mutation rate of ∼3e^−10^ events per site per generation (30), the mean divergence between nonmutator isolates of ∼5 to 20 mutations (the value of the estimator π [see below]) would indicate more than a thousand generations of neutral divergence. Alternatively, selection could be involved to make such an accumulation of mutations in a shorter time frame.

**TABLE 2:** Mutational events occurring in E. coli clones isolated from three extraintestinal infections^*a*^

Patient	No. of mutational events								
	Total	Large deletions	Intergenic		Genic				
			Indels	SNPs	Indels		SNPs		
					In frame	Frameshift	Nonsense	Nonsynonymous	Synonymous
11	36	5	0	4	1	4	2	15	5
13	65	3	4	3	2	19	4	27	7
17	214	35	10	26	3	24	6	94	22

All	315	43	14	33	6	47	12	136	34

a Deletions of more than 50 bp were considered large. Indels, small insertions and deletions; SNPs, single nucleotide polymorphisms.

### Structure of diversity using point mutations.

The point mutations can give us a more quantitative description of the population diversity using simple population genetic metrics. By excluding the mutators, we first calculated the Watterson estimator (θ_w_), which reflects the number of polymorphic sites (31), as well as the nucleotide diversity (π). We obtained values of 27, 11.4, and 6.1 for θ_w_ for patients 17, 13, and 11, respectively. In the same order, π values were equal to 20.5, 9.1, and 4.9. The normalized difference between the two estimates (θ_w_ − π), named Tajima’s *D* (32), can further be computed to infer if the evolutionary process is mostly controlled by genetic drift. Tajima’s *D* estimates were −1.4, −1.3, and −1.3 for patients 17, 13, and 11, respectively. Negative values of *D* are associated with a signature of selection. However, the obtained values do not statistically differ from zero and are insufficient on their own to argue in favor of selection versus genetic drift as the force dominating the evolutionary process at play.

Another approach to look for genomic traces of selection consists of focusing only on point mutations found in coding regions and comparing the rates of nonsynonymous and synonymous mutations. The ratio of the two latter weighted by the reference isolate codon usage bias gives us the *K_a_*/*K_s_* ratio. For patients 17, 13, and 11, excluding mutators, as their impact may be confounding (33), we obtained values of 5.1 and 5.46 and an infinite value (with *K_s_* being equal to 0 in the absence of synonymous mutations as is the case for isolates from patient 11), respectively. These large values can this time be interpreted as a clear sign of positive selection.

**FIG 2:**
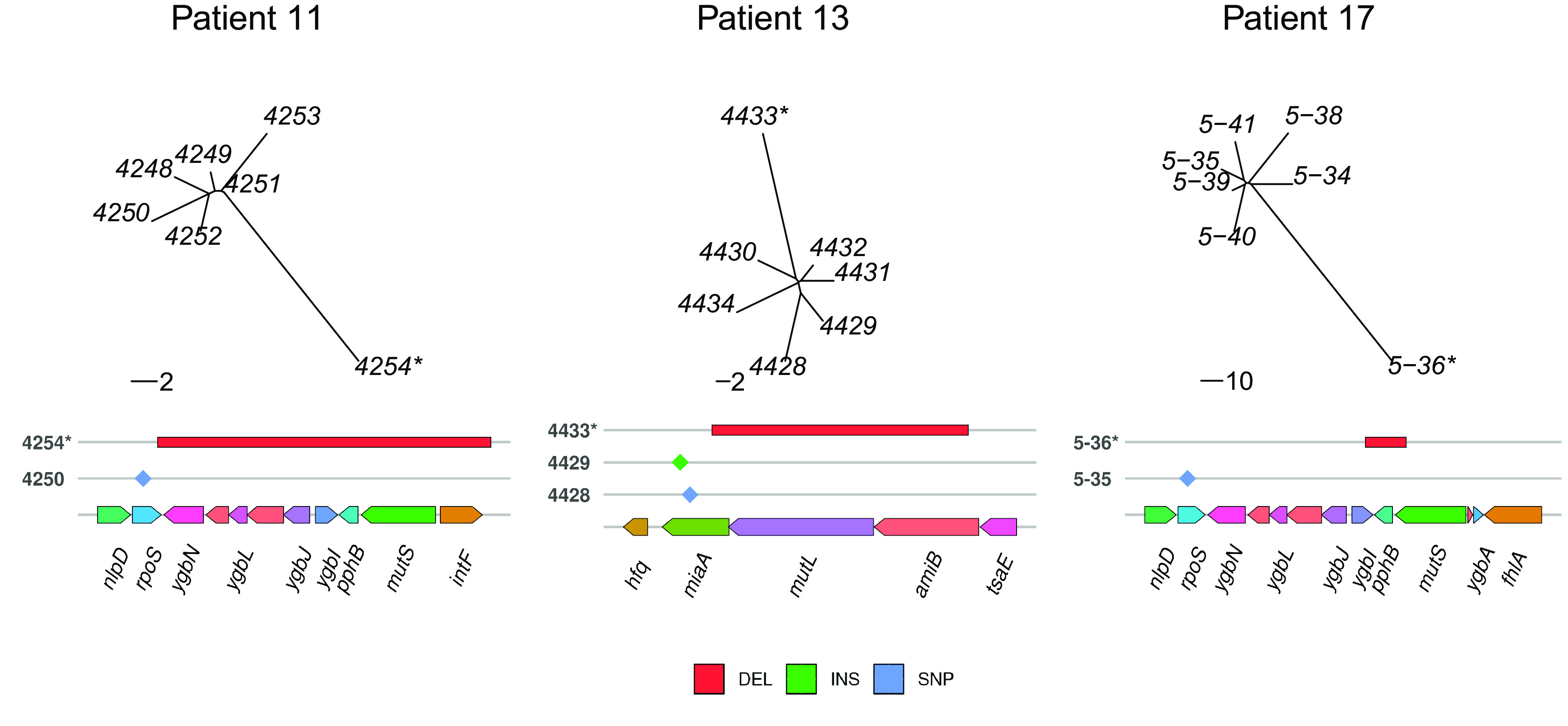
Unrooted trees of isolates from the three patients and molecular mechanism of the mutator genotype. The trees were constructed with the mutation presence/absence matrix using neighbor joining. Below each tree is a representation of the genomic deletion event giving a mutator phenotype, and the convergent mutations observed for some isolates are represented by colored diamonds. The scale bars below the trees are the numbers of substitutions. * indicates a mutator. DEL, deletion; INS, small insertion; SNP, single nucleotide polymorphism.

Finally, we built an unrooted tree using neighbor joining on the mutation presence/absence matrix for each patient (Fig. 2). Besides the long branches observed for the mutator isolates, the star-shaped phylogeny indicates radiation with rapid diversification of the isolates, presumably following a bottleneck.

### Mutator genotype and contribution to diversity.

Within each patient, a mutator was single-handedly responsible for about 30% to 50% of the overall genetic diversity. We were able to identify the genetic basis of the mutator phenotype in all three cases. As expected, they are mutations in the MMR pathway. In patient 17, a 1,478-bp deletion truncates the last 399 nucleotides of the *mutS* coding sequence in the mutator genome; it is known in the literature that such deletions increase the mutation rate by more than a hundredfold in E. coli (34). This *mutS* gene is fully absent in the patient 11 mutator due to a 17,491-bp deletion. Similarly, in the case of patient 13, we saw a deletion of a 3,265-bp genomic segment encompassing *mutL*. Mutations in the MMR mainly increase the number of single nucleotide variants, and ours are no exception as they make up to 57%, 52%, and 38% of the point mutations in patients 17, 11, and 13, respectively. An MMR defect leads to a strong increase in the number of transitions (35), so we compared the numbers of transitions and transversions between the mutator strains and the other isolates in each patient. The association of the mutator phenotype with the substitution type was significant for patient 11 (*P* = 0.002 by a Fisher exact test) and patient 17 (*P* < 0.001 by a Fisher exact test) but inconclusive for patient 13 (*P* = 0.186 by a Fisher exact test).

### Within- and between-patient convergence.

In our study, within-population mutational convergence is defined as the presence of independent mutations in the same genes across multiple isolates of a single patient. This phenomenon is a sign of adaptation, and experimental evolution has revealed that when genes are targets of adaptation, many mutations in the genes may be adaptive, even in essential genes that are highly constrained (36). This gene-level convergence shows that the population is large enough to drive several beneficial mutations simultaneously to a detectable frequency, a phenomenon named clonal interference (37). In the three populations under study, we could find multiple signs of such convergence. We found genes independently mutated more than once 23, 8, and 4 times in isolates from patients 17, 13, and 11, respectively (Table 3). These convergent mutations represent between 50% and 80% of the total number of mutations in each patient, with the mutator excluded. These values are higher than what was reported in a previous study of *in vitro* experimental evolution under strong selective pressure (11).

**TABLE 3:** Mutational convergence observed at the gene level^*a*^

Gene	Function(s)	Mutation type(s)	No. of mutational events			
			Patient 11	Patient 13	Patient 17	Total
*rbsR*	RbsR DNA-binding transcriptional repressor	DEL, SNP, INS	2	6	1	9*
*putA*	Fused PutA transcriptional repressor/proline dehydrogenase	SNP, Large_DEL	0	4	4	8*
*glpD*	Glycerol-3-phosphate dehydrogenase, aerobic	SNP, DEL	0	6	1	7*
*rpoS*	RNA polymerase, sigma S (sigma 38) factor	SNP, DEL, Large_DEL	2	3	1	6*
*ydcI*	Putative transcriptional regulator LysR type	SNP, INS, Large_DEL	0	0	6	6
*fimH*	Minor fimbrial subunit, d-mannose-specific adhesin	SNP, DEL	4	1	0	5*
*acrB*	AcrB RND-type permease	SNP, DEL, Large_DEL	2	1	1	4*
*hupA*	Transcriptional dual regulator HU alpha (HU-2)	DEL, SNP	0	1	3	4*
*putP*	Proline:Na^+^ symporter	SNP, Large_DEL	0	0	4	4
*aldA*	Aldehyde dehydrogenase A, NAD linked	SNP, Large_DEL	0	0	3	3
*fliC*	Flagellar biosynthesis, flagellin, filament structural protein	SNP	1	2	0	3*
*fnr*	FNR DNA-binding transcriptional dual regulator	SNP, Large_DEL	0	2	1	3*
*frsA*	Fermentation/respiration switch protein	SNP, Large_DEL	0	0	3	3
*glnL*	NtrB	SNP, Large_DEL	0	2	1	3*
*miaA*	tRNA(i6A37) synthase	SNP, INS, Large_DEL	0	3	0	3
*paoC*	Aldehyde dehydrogenase:molybdenum cofactor-binding subunit	SNP, Large_DEL	0	0	3	3
*tehA*	TehA TDT transporter	SNP, Large_DEL	0	0	3	3
*actP*	Acetate/glycolate transporter	SNP, Large_DEL	0	0	2	2
*agp*	3-Phytase/glucose-1-phosphatase	SNP, Large_DEL	0	0	2	2
*alaE*	l-Alanine exporter	DEL, Large_DEL	0	0	2	2
*araA*	l-Arabinose isomerase monomer	SNP, Large_DEL	0	1	1	2*
*cueO*	Multicopper oxidase with role in copper homeostasis	INS, SNP	1	0	1	2*
*dacA*	d-Alanyl-d-alanine carboxypeptidase, fraction A, penicillin-binding protein 5	INS	0	0	2	2
*ecpD*	Hypothetical protein	SNP, Large_DEL	0	1	1	2*
*flhA*	Flagellar biosynthesis protein FlhA	INS, Large_DEL	0	1	1	2*
UNASSIGNED_04756	Hypothetical protein	SNP	0	0	2	2
UNASSIGNED_04764	Hypothetical protein	SNP, Large_DEL	0	0	2	2
UNASSIGNED_04776	Hypothetical protein	DEL, Large_DEL	0	0	2	2
*fryA*	Fused PTS enzymes; Hpr component, enzyme I component, enzyme IIA component	SNP, Large_DEL	0	0	2	2
*ilvB*	Acetohydroxybutanoate synthase/acetolactate synthase	SNP	0	1	1	2*
*mreB*	Longitudinal peptidoglycan synthesis/chromosome segregation-directing complex	SNP	1	0	1	2*
*mscK*	Potassium-dependent mechanosensitive channel MscK	SNP, Large_DEL	1	0	1	2*
*mutS*	MutHLS complex, methyl-directed mismatch repair	Large_DEL	1	0	1	2*
*pphB*	Protein-tyrosine-phosphatase/phosphoprotein phosphatase	Large_DEL	1	0	1	2*
*rpsH*	30S ribosomal subunit protein S8	SNP	0	0	2	2
*sstT*	Serine/threonine:Na^+^ symporter	SNP	0	0	2	2
*tbpA*	Thiamine ABC transporter—periplasmic binding protein	SNP, Large_DEL	0	0	2	2
*thiP*	Thiamine ABC transporter—membrane subunit	DEL, Large_DEL	0	0	2	2
*yafL*	Putative lipoprotein and C40 family peptidase	SNP, Large_DEL	0	0	2	2
*ydeO*	YdeO DNA-binding transcriptional dual regulator	DEL, Large_DEL	0	0	2	2
*yedW*	Putative DNA-binding response regulator in two-component system with YedV	SNP, Large_DEL	1	0	1	2*
*yfeO*	Putative ion channel protein	SNP, Large_DEL	0	0	2	2
*ygbI*	Putative DNA-binding transcriptional regulator, DEOR type	Large_DEL	1	0	1	2*
*ynfF*	Oxidoreductase subunit	SNP, Large_DEL	1	0	1	2*

a The genes are ordered by the total number of mutational events recovered. Gene names starting with UNASSIGNED are detected coding DNA sequences (CDSs) that could not be assigned to a known gene. The asterisks in the rightmost column indicate between-patient convergence. Insertions and deletions larger than 50 bp were considered large. Large_DEL, deletion larger than 50 bp; DEL, deletion; INS, insertion; SNP, single nucleotide polymorphism; PTS, phosphotransferase system.

While the above-described convergence between isolates from the same infection was not unexpected, it was interesting to see the appearance of convergence between isolates from different patients. This could involve genes for which convergence was already observed within patients or genes mutated only once per patient in more than one patient. We found 22 genes targeted by such between-patient convergence. For instance, *putA* was mutated 4 times in both patients 17 and 13, whereas *araA* was found to be mutated in only one isolate from each of these patients (Table 3). Some of the loci involved in these convergences are discussed individually below.

### Mutator and convergence.

In patients 11 and 13, the emergence of the mutator is likely due to the deletion of a gene involved in the MMR that encompasses genes also involved in the convergences mentioned above. In the case of patient 11, the deletion of *mutS* in the mutator extends to *rpoS*, a gene independently mutated in another isolate (Fig. 2). Likewise, the gene *miaA*, in which an SNP and a 1-bp insertion were recovered, is also affected by the deletion of *mutL* in patient 13 (Fig. 2). This could indicate that the mutations generating the mutator phenotype are under first-order selection in these two patients. This type of coincidental selection may favor the emergence of the mutator at a high frequency.

In all three patients, some of the mutations involved in convergence were also recovered in mutator clones: a *fimH* mutation was found in the patient 11 mutator isolate; a frameshift in *rpoS* as well as a mutation in *fnr* and in a gene of unknown function were found in patient 13; and finally, 15 mutations were found in the mutator isolate from patient 17 in genes such as *putP*, *acrB*, *alaE*, and *actP*. These mutations are therefore good candidates to explain the increased frequency of the mutator allele in the population by second-order selection. Both first- and second-order selection could happen at the same time in the mutator isolates, and additional work is needed to go further on this question.

### Intrinsic extraintestinal virulence assessed in a mouse model of sepsis.

To characterize the impact on virulence of the mutations that we identified, we assessed the extraintestinal intrinsic virulence of the isolates using a well-validated mouse model of sepsis (16, 17). Ten mice were injected with a culture of one E. coli isolate, and the lethality and time of death were recorded. We defined three categories, as follows: killer strains, which kill 8 or more out of 10 mice, and nonkiller strains, which kill 2 or fewer mice, with anything between being called intermediate killer (17).

**FIG 3:**
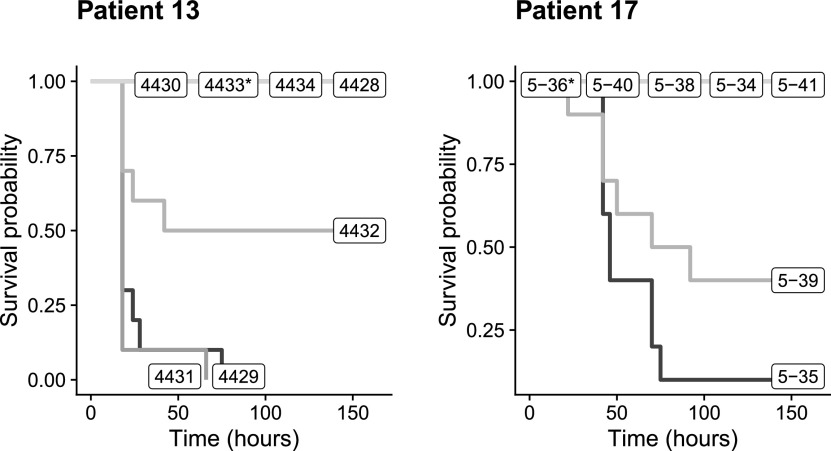
Mouse survival curves after infection by isolates from patients 13 and 17. All surviving mice were sacrificed after 164 h. * indicates a mutator.

While the A phylogroup isolates of patient 11 did not kill any mice, as was expected (16, 17), the isolates of patients 13 and 17 surprisingly exhibited a wide range of virulence ranging from killer to nonkiller phenotypes in the two patients: 29% and 14% of the isolates, respectively, displayed a killer phenotype, and 14% displayed an intermediate-killer phenotype, with the other isolates being nonkiller strains (57 and 72%, respectively). Among the isolates exhibiting a killer phenotype, the ones from patient 13, which belong to the B2 phylogroup, were more virulent, as they killed a majority of the mice in 18 h or less, than the A phylogroup isolates of patient 17, which killed the mice in about 2 days (16) (Fig. 3). In both patients, the mutators were nonkillers.

We then performed association tests between the mortality mouse phenotype of the isolates and the presence of the various mutations showing signatures of within-patient convergence to try to identify the effect of single mutations. No significant association was observed when correcting for phylogeny. This could be due to the small sample size but might also suggest that virulence is a complex trait that can be modulated by several mutations acting together.

### Convergence case studies.

Globally, convergent mutations affected genes involved in global regulation, metabolism, and adhesion/motility functions (Table 3). Below, we dive deeper and present a detailed analysis of some of the convergent mutations found in this study.

### (i) *rpoS*.

We first focused on the *rpoS* mutations, as we have shown in our previous work distinct levels of RpoS in coexisting isolates in extraintestinal infections (21). The RpoS protein is an alternative sigma factor playing a central role in gene expression regulation during the stress response. The regulation of *rpoS* expression happens at many levels: its transcription, translation, stability, and activity regulation are all finely tuned to induce an adapted stress response (38). In the three infections, we recovered 6 independent mutational events. An SNP and a C-terminal deletion were present in patient 11; two frameshifts causing deletions and an SNP were present in patient 13; and finally, a single SNP was detected in patient 17. Moreover, the gene coding for MiaA, a tRNA modification enzyme necessary for potent *rpoS* expression, was also mutated independently in three isolates from patient 13. These results led us to think that a decrease of *rpoS* expression is under positive selection in extraintestinal infections.

We explored further the functional consequences of the D118Y mutation present in the genome of patient 17 isolate 5-35. This missense mutation is localized in the most conserved region of RpoS important for binding to the RNA polymerase core (39), and a mutation of aspartic acid 118 has already been reported and characterized in a clinical isolate of Salmonella enterica (40). In the isolate 5-35 genetic background, we used allelic exchange to generate a strain bearing the wild-type *rpoS* allele (*rpoS*_WT_), a full deletion mutant (Δ*rpoS*), and a strain with the D118Y allele reinserted (*rpoS*_D118Y_) as a control. The red, dry, and rough (rdar) morphotype and resistance to H_2_O_2_, both of which have been linked to RpoS expression (21), indicated that the *rpoS*_D118Y_ mutant had an intermediate effect between the *rpoS*_WT_ and Δ*rpoS* strains (see Fig. S2 in the supplemental material). In our mouse sepsis model, the strain bearing *rpoS*_WT_ or any of the two mutants had the same killer phenotype as the one previously observed in the 5-35 isolate. When inoculated into mice in competition in equivalent proportions two by two or all three together, we saw no difference in survival, and the proportion of each bacterial isolate in the spleen of the mice was the same as that in the inoculum (Fig. S3). These results indicate that substitution D118Y reduces RpoS activity but has no effect on the sepsis phenotype, as was suggested by a previous study of 82 natural isolates that showed no correlation between the *rpoS* alleles and the killer phenotype (41). In addition, the complementation of a truncating RpoS mutation in an isolate from the study showing within-household evolution did not modify the mouse sepsis phenotype (26).

10.1128/mSphere.01176-20.1 FIG S1 Experimental design. A sample is obtained from the patient at the infection site and plated on an appropriate selective medium. Seven isolated E. coli colonies are then randomly selected for further analysis. One of them (in blue) will be sequenced using Illumina and Nanopore technologies in order to obtain a high-quality reference. In parallel, all the isolates (one example being represented in green) are sequenced using Illumina. The reads obtained are then mapped against the *de novo*-obtained reference, and the genetic variations are identified for further analysis. Download FIG S1, PDF file, 0.2 MB. Copyright © 2021 Bridier-Nahmias et al. 2021 Bridier-Nahmias et al. This content is distributed under the terms of the Creative Commons Attribution 4.0 International license.

10.1128/mSphere.01176-20.2 FIG S2 *rpoS* mutant rdar morphotypes and H_2_O_2_ resistance of E. coli isolate 5-35 bearing a wild-type *rpoS* allele (*rpoS*_WT_) or the mutated *rpoS*_D118Y_ allele or deleted for *rpoS* (Δ*rpoS*). (A) The rdar morphotype is dependent on the activity of RpoS. Here, the *rpoS*_D118Y_ allele confers an intermediate phenotype compared to the *rpoS*_WT_ and Δ*rpoS* alleles. (B) Resistance of E. coli to hydrogen peroxide is mediated by RpoS-dependent expression of peroxiredoxin and catalases. Here, anew, the *rpoS*_D118Y_ mutant displays an intermediate phenotype. Download FIG S2, PDF file, 0.05 MB. Copyright © 2021 Bridier-Nahmias et al. 2021 Bridier-Nahmias et al. This content is distributed under the terms of the Creative Commons Attribution 4.0 International license.

10.1128/mSphere.01176-20.3 FIG S3 *rpoS* mutant competition in inoculated mice. (A) Mouse survival curves after inoculation of different combinations of 5-35 isolates bearing wild-type (WT) and mutant *rpoS* alleles. D118Y is *rpoS*_D118Y_, DEL is Δ*rpoS*, and WT is *rpoS*_WT_. (B) Colonies visible after plating on agar supplemented with Congo red in a spleen extract from a mouse infected with the 5-35 *rpoS*_WT_ and Δ*rpoS* isolates in competition. Colonies bearing the *rpoS*_WT_ allele are recognizable by their rdar morphotype (homogeneous red). Download FIG S3, PDF file, 0.6 MB. Copyright © 2021 Bridier-Nahmias et al. 2021 Bridier-Nahmias et al. This content is distributed under the terms of the Creative Commons Attribution 4.0 International license.

### (ii) *rbsR*.

The gene *rbsR* was found mutated in all three patients and up to 6 times in patient 13. It codes for a LacI-type repressor of the *rbsDACBK* operon, mainly responsible for the transportation and phosphorylation of d-ribose. In each patient, at least one mutation disrupts the coding phase of *rbsR*; this could indicate that constitutive expression of the operon is selected for in our isolates. In addition, an *rbsR* missense mutation was identified in one patient suffering from a hepatic abscess and septicemia in our previous work (21) as well as in a patient from the within-household evolution study reported by Kisiela and colleagues (26). This kind of evolutionary pattern is not unheard of. The *rbsDACBK* operon was shown to be an early target of adaptation in Tenaillon et al.’s long-term evolution experiment (42). We have observed *in vivo* the constitutive activation of other inducible pathways in the experimental adaptation of E. coli in the digestive tract of mice. In a first experiment, we observed that the inactivation of the repressor DgoR leads to faster growth in galactonate minimal medium (43). In another experiment, *lacI*, the gene coding for the repressor of the lactose operon, was also selected for inactivation in strains evolving in the gut of lactating mothers and their pups (44). Moreover, *in vivo* studies in mice by another group showed that genes involved in sugar catabolism and sugar transport, especially deoxyribose, are directly linked to the colonization of the gut but also to extraintestinal virulence (45, 46). Taken together, these data point to the major role of sugar metabolism in E. coli adaptation and virulence.

### (iii) *putA*-*hupA*.

The utilization of proline as a source of carbon and nitrogen requires a two-gene operon composed of *putA* and *putP*, coding for the proline utilization flavoenzyme and the proline/sodium symporter, respectively. In the isolates from patients 13 and 17, we found 8 mutations in *putA* (4 each) and 4 mutations in *putP* from patient 17, suggesting that modifications in the proline uptake and degradation pathway are under selection. In these patients, we also found one and three mutations in *hupA* and one mutation in *hupB* in patient 17, always in association with the mutations in *putA* (*P* = 0.027 by a Fisher exact test). HupA and HupB are the two subunits of the histone-like protein HU that regulates DNA topology and plays a pleiotropic role in transcription regulation (47).

As mutations in *putA* without mutations in *hupA* or *hupB* are present in patient 13, this suggests, provided that selective pressures are identical in both infections, that *putA* mutations predated the selection of *hupAB* mutations. As *hupA* inactivations have been described as deleterious for long-term survival or survival under stress conditions (47, 48), this further suggests that *putA* modifications may alleviate the cost of *hupA* mutations.

However, the potential role of these mutations in the adaptive infectious process is unclear. Indeed, proline oxidation into glutamate catalyzed by PutA, aside from providing energy to the cell, increases oxidative stress resistance (49). Further experiments are clearly needed to decipher the significance of these observed mutations.

### (iv) *fimH* and *fliC*.

The gene *fimH* codes for the most distal subunit of the type 1 fimbria adhesion organelle and is expressed in an operon that also comprises the *fimA*, *fimF*, and *fimG* genes. The type 1 fimbriae enhance the virulence of a strain for the urinary tract and are generally involved in the binding of bacteria to the mucosa (50). Polymorphisms in *fimH* through mutations can enhance or reduce the adhesion phenotype and be an important factor in the transition between commensality and pathogenicity (28, 51). It was shown previously that three residues of FimH in particular were able to regulate the transition between low- and high-affinity conformations for mannose and were positively selected during urinary tract infection (29). In patient 11, strong convergence was observed in this gene as it was found to be mutated four times independently, with one isolate exhibiting two mutations. Indeed, mutations L133V and V150L were found together in isolate 4250, while mutations Q62K and T179A were found in isolates 4249 and 4254, respectively. In the case of patient 13, a 36-bp deletion in the mannose-binding domain near the N-terminal region is present in isolate 4431.

In a very similar fashion, the E. coli flagellin protein encoded by *fliC*, the major component of the flagellum apparatus, is obviously crucial for cell motility but is also known to be involved in innate and adaptive immune responses (52). Moreover, Girón and colleagues showed for the first time in the early 2000s that the flagellum had adhesive properties and that *fliC* mutants were impaired in adherence (53). We found three independent nonsynonymous point mutations in *fliC* in three isolates, one from patient 11 and two from patient 13.

Interestingly, the gene *lrhA*, coding for a global regulator of E. coli genes that, among other effects, represses the expression of type 1 fimbriae and flagella (54), has been found mutated in isolates from both households in the above-described study showing within-household evolution (26). Complementation of the mutation in a mouse sepsis assay showed that the mutation increased the intrinsic virulence of the strain (26).

On the whole, these data bring solid arguments in favor of mutations involved in adherence and motility functions being under strong selective pressure during infections.

### Conclusion.

In this study, we aimed to characterize the adaptation of E. coli during acute extraintestinal infections. To this end, we have sequenced isolates, one of which was a strong mutator, from three different infections from various body sites using Illumina and Oxford Nanopore technologies in order to obtain high-quality genomic data. These data were processed using well-known bioinformatics tools and in-house scripts, giving a comprehensive catalogue of the mutations present in the collected isolates. We uncovered a high level of convergence both between and within the three infections as mutations found in genes hit multiple times represented more than 40% of the total. This suggests that adaptation is at play in these infections and that sufficient levels of diversity can be generated efficiently in E. coli in environments as diverse as the peritoneal fluid, the kidney, and the cerebrospinal fluid.

The major limitation of our study lies in the lack of a dynamic time frame for the occurrence of the observed mutations. Based on the limited genetic diversity and the absence of selection signals that we observed previously in the gut of a healthy donor (55), we attributed the large diversity and the signatures of selection detected in the present study to the infectious process. It is, however, possible that some of the genomic footprints that we observed initiated in the gut environment or that some mutations may have emerged on plates after the infection (56). For the latter point, we minimized the risks by using clones that had been subcultured only twice. For the former, sequencing samples simultaneously from the gut and the infective site and ideally with several sampling times therefore appears to be the next objective. This would, however, be quite challenging for two main reasons. First, despite their important burden at the population level, E. coli extraintestinal infections per individual are rare. Consequently, a large effort would be needed to sample in a prospective way a population of healthy individuals in order to find only a few with an infection. Second, for infected individuals, while sampling the infective site is often one of the first medical gestures, it is almost immediately associated with antibiotic treatment, before feces can be sampled. A more pragmatic alternative would be to perform these studies of diversity in many healthy individuals and in many infections to see how typical is the pattern that we observed here.

Our data illustrate the early emergence of mutator alleles under natural conditions. Indeed, the mutators did not diverge too much, and we could hope to identify the background mutations that drove them to high frequencies. As several mutations involved in convergence were also recovered in the mutator isolates, some of them might have participated in their increased frequency. Interestingly, in two out of three cases, the mutation conferring the mutator phenotype could be adaptive by itself. In both cases, it is due to a large deletion that encompasses multiple genes that were suspected to be the target of selection by themselves as they were mutated in other isolates. The mutator may then have emerged as a by-product of selection acting on the neighboring genes. This early selection of mutator isolates could be coincidental in each infection, but their relatively low frequency in the population (14%) is in contrast to their important contribution to diversity, especially when considering point mutations. These mutators may represent an important reservoir for diversity and thus contribute greatly to the evolvability of the population.

## MATERIALS AND METHODS

### Human ethics statement.

All the sampling procedures on the infected patients were performed in the course of clinical diagnosis. No additional procedure was performed on the patients for the present study. The study was approved by the institutional ethics committee (Comité de Protection des Personnes, Hôpital Saint-Louis, Paris, France, approval number 2004-06). The participants were informed of their role in the study, and written informed consent was provided by the study participants.

### Isolation of clone isolates.

Three adult patients with severe extraintestinal E. coli infections were studied (21): patient 11 had neurosurgery postoperative infection of the cerebrospinal fluid, patient 13 suffered from pyelonephritis, and patient 17 had peritoneal fluid infection. In all cases, E. coli alone was recovered. Each patient was infected by a single clone. For each patient, seven isolates were randomly selected after seeding the pathological sample on blood agar (patients 11 and 17) or cysteine lactose electrolyte-deficient (patient 13) plates. The isolates were subcultured twice and stored in glycerol at −80°C.

### Strain typing.

Classical epidemiological typing using the Web interface of the Center for Genomic Epidemiology and ClermonTyper (57) confirmed that each patient was infected by isolates of a single clone defined according to the phylogroup, the sequence type (Warwick University and Institut Pasteur schemes) (58), the serotype (59), and the *fimH* type (60) (Table 1).

### Sequencing and assembly.

All the isolates were sequenced using Illumina sequencing. All the isolates from patient 13 as well as 7 of the isolates from patient 17 were sequenced in 50-bp single reads on a HiSeq 2000 instrument. Isolate 5-36 from patient 17 was sequenced in 100-bp single reads later on equivalent equipment. Finally, the isolates from patient 11 were all sequenced in 100-bp paired-end reads on a HiSeq 4000 sequencer.

For each patient, one nonmutator isolate was chosen as a reference and was sequenced on the Oxford Nanopore Technologies MinION platform using an R9.4 flow cell, and samples were prepared using kits LSK-108 and NBD-104 for library preparation and barcoding. A hybrid assembly strategy with Unicycler software V0.4.4 (61) provided high-quality assembly on which to base further genomic analysis.

### Genomic analysis.

For each patient, the Illumina reads for each isolate and the corresponding reference generated as mentioned above were independently fed to Breseq V 0.39.0 (62). Breseq outputs were processed using an in-house-made pipeline. All the code is available as an R notebook in a repository at https://github.com/A-BN/acute_infect_evol. A table containing all the recovered mutations is available in Data Set S1 in the supplemental material as well as at an interactive webpage at https://a-bn.github.io/acute_infect_evol/2020-all_mutations.html.

10.1128/mSphere.01176-20.4 DATA SET S1 Tab-separated value (tsv) table containing all the recovered mutations with their metadata. The column names are written as explicitly as possible. Download Data Set S1, CSV file, 0.6 MB. Copyright © 2021 Bridier-Nahmias et al. 2021 Bridier-Nahmias et al. This content is distributed under the terms of the Creative Commons Attribution 4.0 International license.

### Mutation rate assay.

Mutation frequencies of the isolates were estimated by monitoring their capacity to generate mutations conferring resistance to rifampicin in triplicates in at least three independent cultures (nine values minimum), as described previously (22).

### rdar morphotype and H_2_O_2_ resistance.

Cells of the mutants of isolate 5-35 from patient 17 were tested by red, dry, and rough (rdar) morphotype and H_2_O_2_ resistance assays, which are dependent on RpoS. For the rdar phenotype, cells were grown on LB agar plates without NaCl (LB0) at 28°C. We determined colony morphology and color using LB0 agar supplemented with Congo red (40 μg · ml^−1^) and Coomassie brilliant blue (20 μg · ml^−1^), as described previously (21, 63). For the H_2_O_2_ resistance assay, cells were grown overnight in LB medium, washed, and resuspended in 0.9% NaCl to a concentration of about 1e^8^ cells · ml^−1^. H_2_O_2_ was added to a final concentration of 5 mM. Aliquots of bacteria were removed at 0, 60, and 90 min, and numbers of viable cells were determined on LB plates.

### *rpoS* allelic exchange in the 5-35 isolate.

Allelic exchange of *rpoS* in the 5-35 isolate exhibiting the *rpoS*_D118Y_ mutated allele was achieved using a two-step Red recombinase-based recombineering procedure (64). Briefly, the *tetAR* resistance module was PCR amplified with 60-mer primers matching in their extremities to the 5′ and 3′ parts of the *rpoS* gene and electroporated into the 5-35 isolate previously transformed by the pKD46 plasmid (65). Recombinant tetracycline-resistant clones were selected and checked by PCR, and the presence of the chromosomal Δ*rpoS*::*tetAR* mutation was confirmed by DNA sequencing. The *tetAR* cassette was then either deleted (5-35 Δ*rpoS*) or replaced by the *rpoS* wild-type allele amplified from the 5-40 isolate (5-35 *rpoS*_WT_) or the original allele of the 5-35 isolate (5-35 *rpoS*_D118Y_) through positive selection on Bochner-Maloy plates of tetracycline-sensitive recombinants (66). The presence of the desired mutations in these strains was confirmed by Sanger DNA sequencing.

### Mouse sepsis assay.

A mouse sepsis model was used to assess the intrinsic extraintestinal virulence of the isolates as described previously by Picard et al. (16). Briefly, for each isolate, 10 outbred female Swiss OF1 mice (3 to 4 weeks old) from Janvier Labs (Le Genest-Saint-Isle, France) received a subcutaneous injection into the nape of the neck of approximately 2 × 10^8^ CFU of stationary-phase bacteria. After inoculation, the mice (which had free access to food and water) were observed for up to 1 week. The time of death was recorded for each mouse. Surviving mice were euthanatized on day 7 by cervical dislocation. In each lethality assay, two control isolates were included: the K-12 MG1655 isolate, which does not kill mice by 7 days postchallenge, and the CFT073 isolate, which shows lethality of ≥80% by 7 days postchallenge (17). Control isolates were prepared under the same conditions as those for the test isolates and injected into 5 mice each. Isolates were categorized as killer or nonkiller if they killed ≥80% or ≤20% of the inoculated mice, respectively. Isolates killing mice at a rate between these two values were categorized as intermediate killers (17).

Competition experiments for *rpoS* mutants were performed by inoculating 5 to 6 mice under the same conditions as the ones described above with a 1/1 ratio of the various mutant and wild-type strains. Bacterial cells were enumerated in the spleens of animals after grinding and sprawl on lysogeny broth plates. The distinction between wild-type and mutant strains was done phenotypically by looking at the rdar morphotype, which is dependent on RpoS activity (63).

### Animal ethics statement.

Animal experiments were performed in compliance with the recommendations of the French Ministry of Agriculture and approved by the French Veterinary Services (accreditation A 75-18-05). All animal experimentation was conducted according to European (directive 2010/63/EU on the protection of animals used for scientific purposes) and National (RD 53/2013) regulations for transport, housing, and care of laboratory animals. The protocol used was approved by the Animal Welfare Committee of the Veterinary Faculty in Lugo, University of Santiago de Compostela (AE-LU-002/12/INV MED.02/OUTROS 04). All efforts were made to minimize suffering.

### Data availability.

The sequences were deposited in the European Nucleotide Archive (ENA) with the accession number PRJEB41453.
