# Supplementary figures and images for: Escherichia coli Genomic Diversity within Extraintestinal Acute Infections Argues for Adaptive Evolution at Play
Source: mSphere. 2021 Jan 6;6(1):e01176-20. doi: 10.1128/mSphere.01176-20 (PMC7845604; doi:10.1128/mSphere.01176-20)

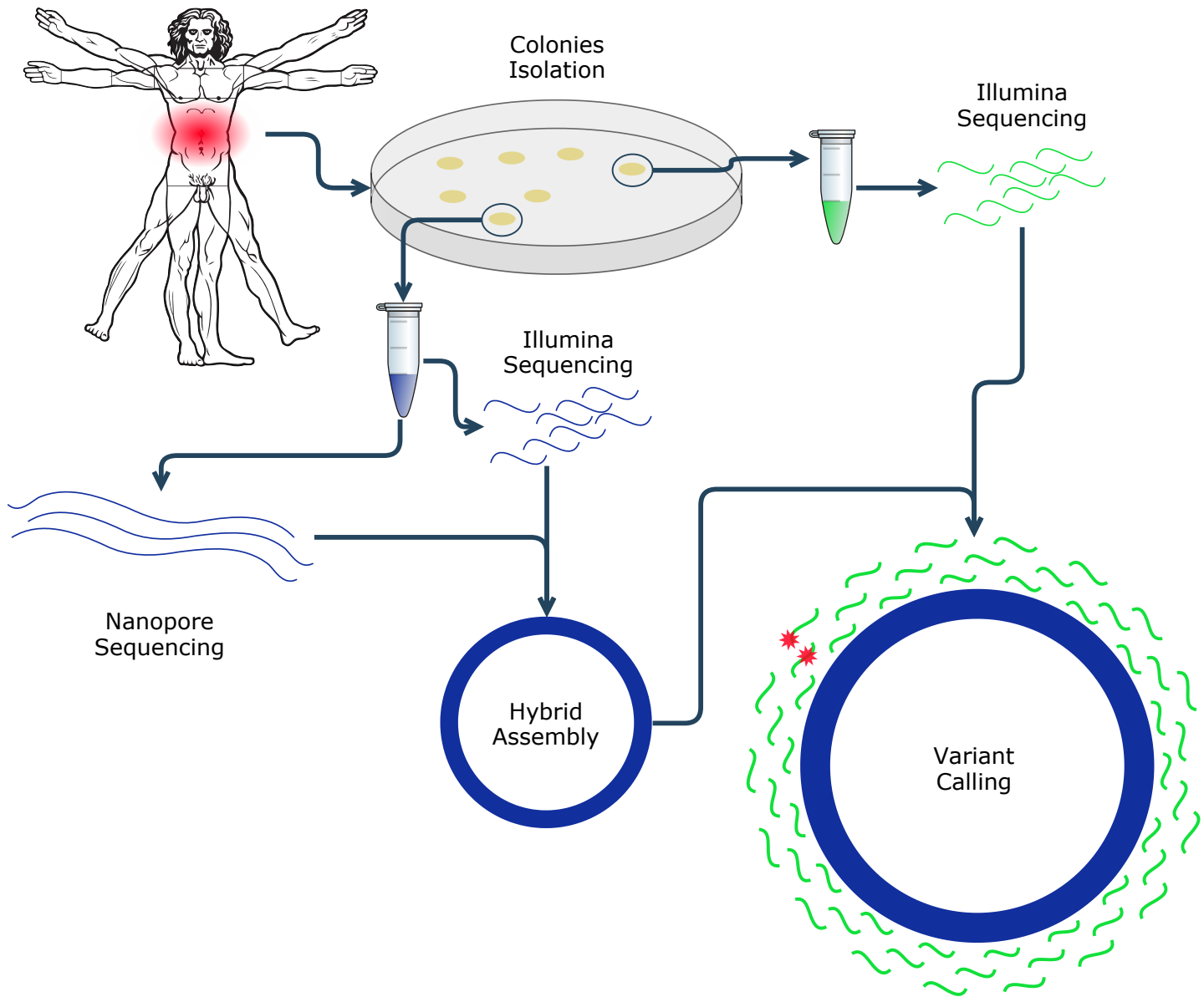

Supplement: FIG S1 [file mSphere.01176-20-sf001.pdf]

**A**

Patient 17 isolate 5-35:

*rpoS*<sub>WT</sub>

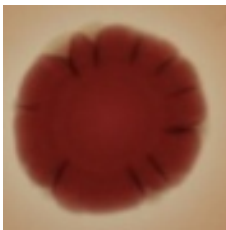

*rpoS*<sub>D118Y</sub>

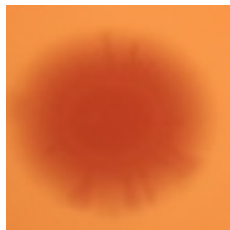

$\Delta$ *rpoS*

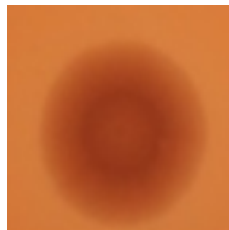**B**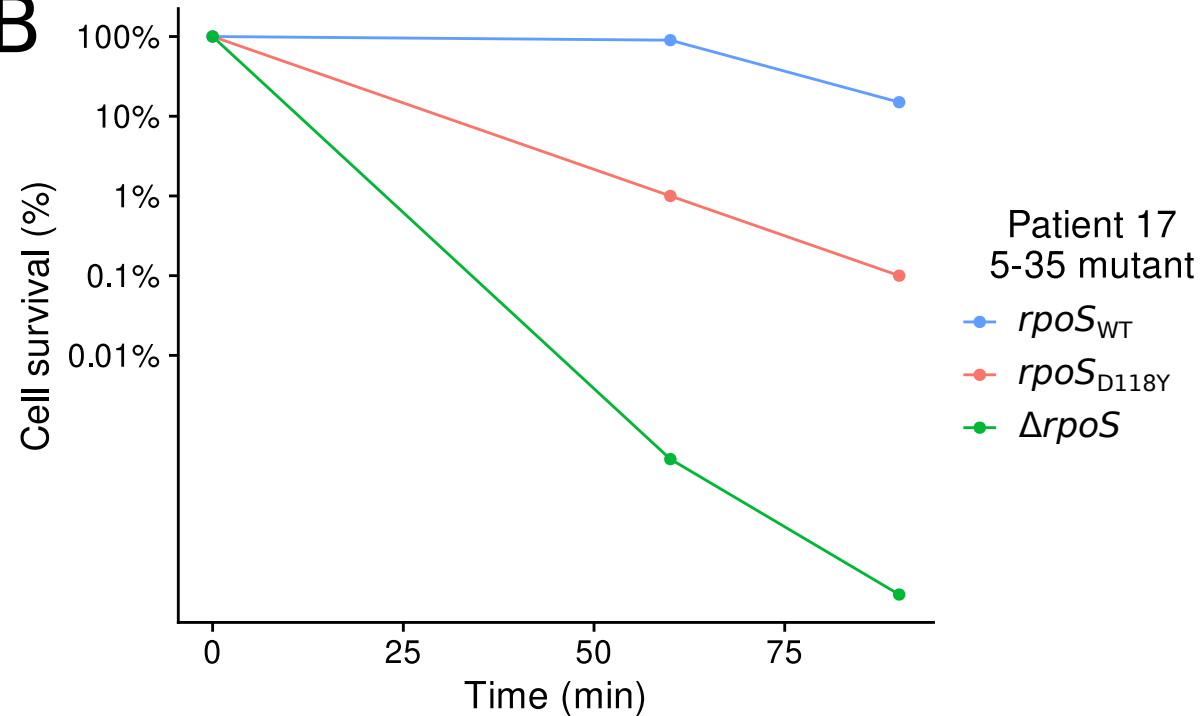

Supplement: FIG S2 [file mSphere.01176-20-sf002.pdf]

**A**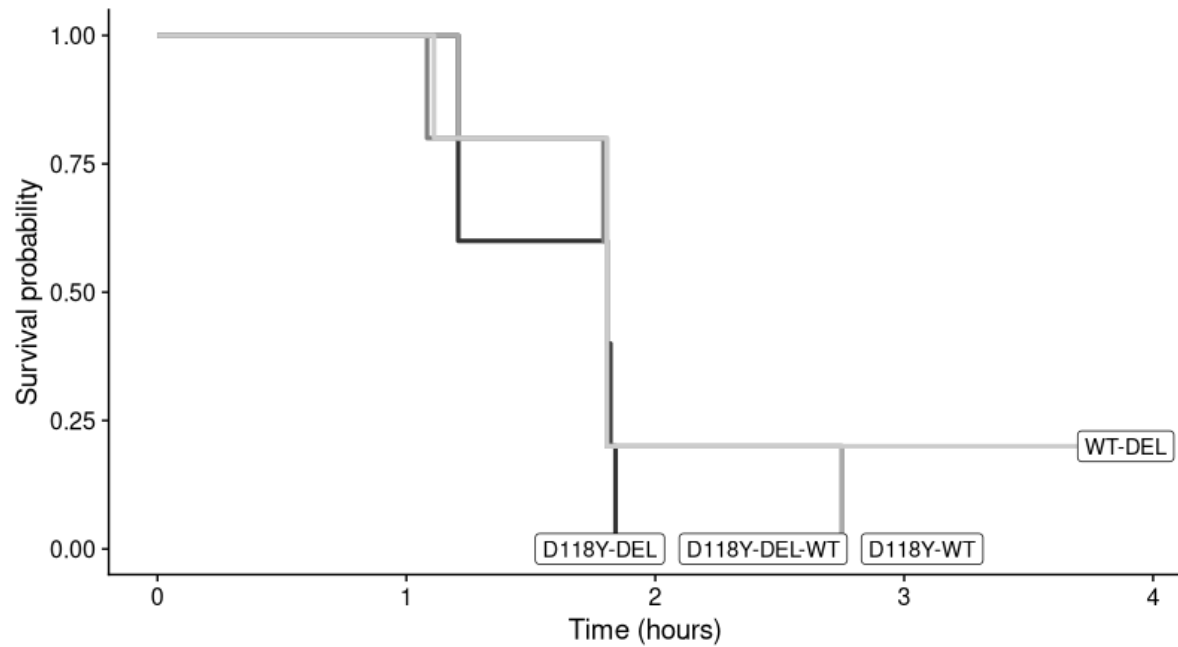**B**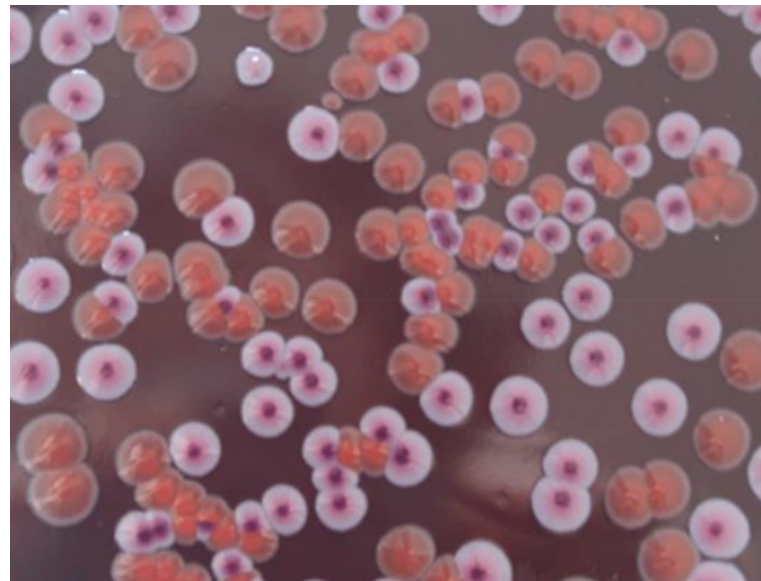

Supplement: FIG S3 [file mSphere.01176-20-sf003.pdf]
